# Uncertainty of laboratory and portable solid particle number systems for regulatory measurements of vehicle emissions

**DOI:** 10.1016/j.envres.2021.111068

**Published:** 2021-06

**Authors:** Barouch Giechaskiel, Tero Lähde, Anastasios D. Melas, Victor Valverde, Michaël Clairotte

**Affiliations:** European Commission – Joint Research Centre (JRC), 21027, Ispra, VA, Italy

**Keywords:** Particle measurement programme **(**PMP), Portable emissions measurement system (PEMS), Diffusion charger (DC), Particle number counter (PNC), Condensation particle counter (CPC), Volatile particle remover (VPR)

## Abstract

In the European Union’s emissions regulations, limits for solid particles >23 nm are applicable for the type-approval and in use compliance of vehicles. Consequently, particle number (PN) systems are used very often for both research and development of engines and vehicles, both in the laboratory and on the road. The technical specifications of the laboratory and portable on-board systems are not the same resulting in different measurement uncertainties. Furthermore, particles, in contrast to gases, can be lost in the transfer lines making comparisons at different sampling locations difficult. Moreover, the size dependent counting efficiency of the systems can result in high discrepancies when the measured particle sizes are close to the decreasing steep part of the curves. The different sampling locations (tailpipe or dilution tunnel) and thermal pretreatments of the aerosol further enhance the differences. The studies on the measurement uncertainty are scarce, especially for the PN systems measuring from 10 nm that will be introduced in the future regulations. This study quantified the uncertainty sources of the PN systems: (i) due to the technical requirements and the calibrations, (ii) due to the unknown particle sizes during measurement, (iii) due to particle losses from the vehicle to the PN systems at the tailpipe or the dilution tunnel, (iv) other parameters needed for the calculation of the emissions, non-related to the PN systems, e.g. flow and distance. The expanded uncertainty of the 23 nm laboratory systems sampling from the dilution tunnel was estimated to be 32%, with 18% originating from the calibration procedures, while of those sampling from the tailpipe 34%. For the 23 nm portable systems measuring on-road the uncertainty was 39%. The values were 2–8% higher for the 10 nm systems.

## Highlights

• Measurement uncertainty of laboratory and portable particle number systems.
• The differences between systems are due to the technical specifications, the unknown particle size and the particle losses.
• The expanded uncertainty, depending on the application, was 32%–42%.

## Abbreviations

acc: accuracy
C: concentration
CE: counting efficiency
CNG: compressed natural gas
CPC: condensation particle counter
CVS: constant volume sampling
D: distance
DPF: diesel particulate filters
ECU: electronic control unit
exh: exhaust
GDI: gasoline direct injection
GMD: geometric mean diameter
GPS: global positioning system
GSD: geometric standard deviation
GTR: Global technical regulation
GUM: Guide to the Expression of Uncertainty in Measurement
HDV: heavy-duty vehicles
ICE: internal combustion engine
L: losses
lin: linearity
NRMM: non-road mobile machinery
OBD: on-board diagnostics
PCRF: particle number concentration reduction factor
PEMS: portable emissions measurement systems
PFI: port fuel injection
PMP: particle measurement programme
PN: particle number
PNC: particle number counter
Q: exhaust flow rate
RDE: real-driving emissions
Ref: reference
TP: tailpipe
UNECE: United Nations Economic Commission for Europe
V: volume
VPR: volatile particle remover
WLTC: worldwide harmonized light vehicles test cycle
ρ: density
σ: relative uncertainty

## 1 Introduction

Particulate matter is a major concern for urban air quality. Vehicles may contribute up to 90% of the particle number concentrations in busy roads (Kumar et al., 2014) which is alarming considering the adverse health effects of exhaust particles (da Silva et al., 2019; de Homdedeu et al., 2020). A major breakthrough in the European Union’s vehicle emissions regulations was the introduction of the solid particle number (PN) methodology in 2011 (Euro 5b) for compression ignition vehicles (diesel) (Giechaskiel et al., 2012b). Later, in 2014 the PN limit was introduced for gasoline direct injection vehicles (Giechaskiel et al., 2019b). In 2013 and in 2017 heavy duty engines and non-road mobile machinery (NRMM) were included, respectively. The PN limits practically forced the use of diesel particulate filters (DPFs) in all diesel vehicles. The laboratory systems were based on the technical specifications drafted by the United Nations Economic Commission for Europe (UNECE) informal group particle measurement programme (PMP) group, and for this reason the laboratory systems are usually called as PMP systems (Giechaskiel et al., 2008b). The PN systems are sampling from the full dilution tunnel with constant volume sampling (CVS). The PMP systems include a volatile particle remover (VPR) and a particle number counter (PNC) that measures particles from approximately >23 nm.

With the introduction of the real-driving emissions (RDE) regulation in 2017, the laboratory limits for the light-duty vehicles have to be respected also on-road under normal operation of use. The limits are considering the measurement uncertainty of the on-board systems (Giechaskiel et al., 2019a), in particular the additional uncertainty of the on-board measurement systems compared to the laboratory ones (called margin or conformity factor). This means that the variability of the emissions of the vehicle due to the environmental conditions, the driving style, and the driving route (under the boundaries defined in the regulation) are not included in the margin and the respective limits. Thus, it is important that the uncertainty of the on-board instruments is well defined in order to avoid wrong decisions on the compliance of a vehicle with the legal limits. A similar on-road approach was introduced in 2021 for heavy-duty vehicles. The additional uncertainty of the portable emissions measurement systems (PEMS) was estimated to be around 50%–60%; roughly 30–35% due to the instrument specifications, and 15–25% due to the different sampling locations (tailpipe for PEMS versus dilution tunnel for PMP systems) (Giechaskiel et al., 2019a). Particles, in contrast to gases, can be lost via diffusion and thermophoresis or agglomerate to form bigger particles in the transfer lines (Giechaskiel et al., 2012a). The measurement uncertainty of PEMS was based on experimental results in one laboratory testing many vehicles, and many laboratories testing one “Golden” vehicle (Giechaskiel et al., 2019a). Theoretical estimations presented in the RDE group meetings were also in agreement with the 50% uncertainty.

Recently, for PMP systems an option of measuring from approximately >10 nm, instead of >23 nm as in the current regulations, was added in the GTR 15 (global technical regulation) (Lahde et al., 2020). The 10 nm PEMS technical specifications were also drafted in order to match the new specifications of the 10 nm PMP laboratory systems. A few experimental campaigns gave differences between prototype 10 nm PEMS and PMP laboratory systems of lower than 40% (Giechaskiel et al., 2019e, 2020a). Another important future change is that measurements directly from the tailpipe will also be allowed for laboratory PMP systems, at least for heavy-duty engines (Martini and Grigoratos, 2021). Thus, a re-evaluation of the measurement uncertainty is necessary for both PMP under laboratory conditions and PEMS measuring on-road.

PN measurements are nowadays a standard procedure for both research and monitoring of vehicle emissions (Bielaczyc and Szczotka, 2007; de Melo and Dias, 2004; Giechaskiel et al., 2018d; Kontses et al., 2020; Maricq et al., 2017; Mishra et al., 2015; Mohanta et al., 2014; Yamada et al., 2015). As with the gaseous pollutants the uncertainty analysis is an important part for all measurement fields, and in particular, a critical aspect for compliance assessment. Most of the studies have focused on gaseous pollutants (Balawender et al., 2016; Joumard et al., 2009; Sherman et al., 2001; Velosa, 1993), while the studies on PN are limited and focused on the laboratory systems at the dilution tunnel (Gilham and Quincey, 2007; Kirchner et al., 2010). Even though the topic of uncertainty has been discussed in atmospheric measurements even for sub-10 nm particles (Kangasluoma et al., 2020), the analysis for the high temperature, dynamic and condensing exhaust aerosol is lacking. Furthermore, to our knowledge, there is no comprehensive analysis of the uncertainty of PN systems, and in particular on the road.

The objective of this study is to calculate typical and maximum (worst case) uncertainties of PEMS and PMP systems with lower sizes of both 23 nm and 10 nm sampling from the dilution tunnel or the tailpipe. Typical here refers to the uncertainty that commercial systems have in practice, while maximum refers to the uncertainty of systems taking into account all flexibilities allowed in the regulations. The term “system” is used for instrument including certain operational parameters, such as minimum dilution, sample residence time and operational temperatures defined in the regulations, but also including the sampling lines. The results will be compared with other theoretical and experimental studies (e.g. inter-laboratory exercises). The results of this study will help regulators assess future permissible tolerances (i.e. differences between PEMS and laboratory PMP systems) and on-road limits taking into account the additional PEMS measurement uncertainty. For researchers, the typical uncertainties will help them to better assess their results and any comparisons between PN systems.

## 2 Materials and methods

### 2.1 Background

The uncertainty of the result of a measurement reflects the lack of exact knowledge of the “right value” of the sample. It is assumed that the result of a measurement has been corrected for all recognized significant systematic effects and that every effort has been made to identify such effects. It is also assumed that deliberate “errors” have been avoided. The result of a measurement after correction for recognized systematic effects is still only an estimate of the value of the sample because of the uncertainty arising from random effects and from imperfect correction of the result for systematic effects.

In the guide to the expression of uncertainty in measurement (GUM), Type A evaluation of uncertainty is defined as the method of evaluation of uncertainty by the statistical analysis of series of observations. Examples of such methodology are repeatability testing or inter-laboratory exercises. The uncertainty can be evaluated by statistical means, if all of the influencing factors can be assessed experimentally. This is the preferred method when the factors influencing the vehicle exhaust sample are investigated. However, this is not always possible in practice due to limited time and resources. In such cases, the uncertainty of a measurement result can be evaluated using a mathematical model and the law of propagation of uncertainty. Type B evaluation of uncertainty is defined as the method of evaluation of uncertainty by means other than the statistical analysis of series of observations. The Type B approach can be used to estimate the uncertainty of PN systems under chassis dynamometer or on-road conditions using as input mainly the technical requirements of the systems described in the relevant regulations (e.g. accuracy, linearity, counting efficiency etc.). Sometimes combinations of Type A and Type B analysis can be conducted; in particular, to confirm that the uncertainty observed applying the Type A approach is equal or lower than the uncertainty calculated applying the Type B approach. If Type An uncertainty is higher than type B uncertainty following the best engineering practices, then the Type B analysis is not complete and/or some of the underlying assumptions are not valid. The following analysis is based on the Type B analysis and follows the principles of our previous uncertainty analysis on NO_x_ (Giechaskiel et al., 2018b).

**Fig. 1:**
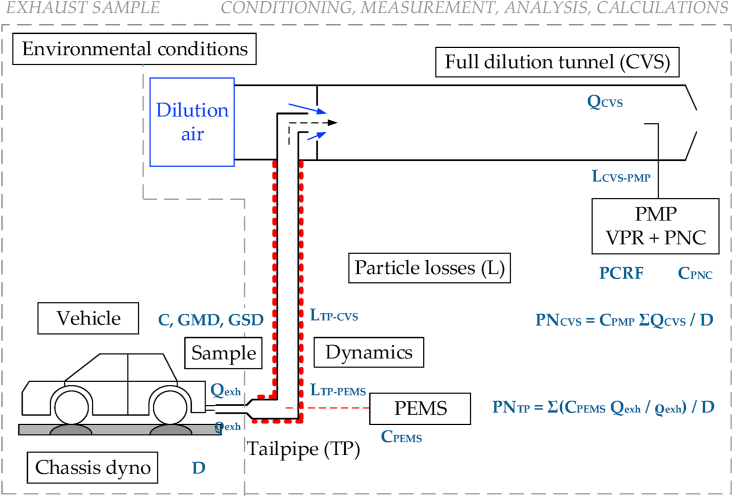
Main influencing factors of the particle number (PN) emissions result. Details in the text. C = concentration; CVS = constant volume sampling; L = losses; D = distance; exh = exhaust; GMD = geometric mean diameter; GSD = geometric standard deviation; PEMS = portable emissions measurement system; PMP = particle measurement programme system; PNC = particle number counter; Q = exhaust flow rate; TP = tailpipe; VPR = volatile particle remover; ρ = density.

Fig. 1 presents a typical experimental setup for the measurement of vehicle PN emissions with a PMP system and a PEMS. The relevant regulations for light-duty or heavy-duty vehicles have only minor differences (such as calibration interval of 12 or 13 months respectively, temperature 350 °C or 300–400 °C respectively) and it is assumed that do not affect the uncertainty analysis described below. The PEMS measures directly from the tailpipe of the vehicle. The PMP instrument is located at the full dilution tunnel (CVS). The main influencing factors of the uncertainty of the PN result are given in frames and are divided in two major areas:

• Area A: Exhaust sample (left part of the figure), which includes the factors that influence the PN size distribution (concentration, size, width) emitted from the vehicle, and
• Area B: Sample conditioning, measurement, analysis and calculations area (right part of the figure) that includes factors directly related to measurement system and method.

A laboratory should ensure that all conditions are within the regulation limits or specifications, avoid errors, and minimize the variability and uncertainty of all influencing factors in both areas. For example, the road load derivation at the chassis dynamometer (Pavlovic et al., 2018), the cooling air flow of the fan in front of the vehicle (Gis et al., 2011), the environmental conditions of the test cell (e.g. temperature, humidity) (Chan et al., 2013; Giechaskiel et al., 2015; Suarez-Bertoa and Astorga, 2018; Valverde and Giechaskiel, 2020), the driver’s style (Gallus et al., 2017; Giechaskiel et al., 2015; Suarez-Bertoa et al., 2019), the vehicle pre-conditioning and soaking time (Srubar et al., 1978), the fuel (Leach et al., 2019; Zhao et al., 2019) and lubricant oil (Amirante et al., 2017; Pirjola et al., 2015; Vaaraslahti et al., 2005) are factors that could influence the particle size distribution that exits from the vehicle (i.e. sample). Not only the particle number concentration (C) will vary, but also the geometric mean diameter (GMD) and the geometric standard deviation (GSD) of the size distribution. Furthermore, volatile nucleation mode particles may exist (Giechaskiel et al., 2020b; Kittelson, 1998) and the chemical composition of the non-volatile particles may also vary (Burtscher, 2005; Maricq, 2007). Ideally, according to the regulation, an instrument would measure only the size distribution of the non-volatile particles. However, due to the technical specifications in the regulation only a specific part of the size distribution is measured (i.e. in current regulations only particles >23 nm). The measurement from the tailpipe needs time aligned signals (exhaust flow rate and particle number concentration) for the correct calculation (Giechaskiel et al., 2019c). The dynamics of the signal can have an influence on the results, in particular when the measurement of the PN and the exhaust flow are done at different location or the response time of the two signals are different (Ajtay and Weilenmann, 2004; Geivanidis and Samaras, 2008; Mahadevan et al., 2016). Furthermore, particle transformations and losses from the tailpipe until the PN system can change the size distribution (Giechaskiel et al, 2012a, 2019c). The existence of volatiles might interfere with the measurement of non-volatile particles (Giechaskiel et al., 2020b). The focus of this paper is on the second area (Area B) and in particular the uncertainty of the instruments (PMP and PEMS). Nevertheless, the sample preparation will also be discussed.

The uncertainty of the PN systems can be further divided in two categories: Uncertainties due to the technical specifications (e.g. calibration accuracy, linearity etc.) and uncertainties due to the methodology and sampling location (e.g. thermophoretic and agglomeration losses differences due to sampling at the tailpipe or the dilution tunnel).

The PN systems (PMP and PEMS) have different technical requirements. A PMP system typically consists of a VPR (volatile particle remover) heated at 350 °C and a PNC (particle number counter) counting particles with 50% detection (counting) efficiency for 23 nm particles or 65% detection efficiency for 10 nm particles, depending on the regulation (Lahde et al., 2020). The detection efficiency is the ratio of the PNC’s and a reference instrument’s particle concentrations when measuring in parallel particles of a specific diameter. The VPR is calibrated for each dilution setting with 30 nm, 50 nm, and 100 nm particles with the PCRF (particle number concentration reduction factor). The mean PCRF of 30 nm, 50 nm, and 100 nm, which is practically the dilution including the average particle losses in the 30 nm–100 nm size range, is used for the calculation of the emissions. For the PMP systems, PNC efficiency and VPR losses are determined separately. The PEMS on the other hand is calibrated as a whole system and has to fulfill specific efficiency requirements (i.e. compared to a reference instrument it must be within 70%–130% when measuring 70 nm particles, 20%–60% for 23 nm) (Giechaskiel et al., 2019a). Details can be found in Appendix.

The assessment of the PN measurement uncertainty has some major differences and difficulties compared to other uncertainty cases (e.g. gases):

• The “ideal” measured concentration by the PN system is not necessarily the actual emissions from the vehicle, as regulations specify lower cut-off and “efficiency curves”.
• The particle size, morphology and chemical composition are not constant and/or known during a test and this results in differences between the PN systems having different penetration curves and response functions for different materials.
• Particle losses and transformations from the vehicle to the PN system can affect the instrument’s inlet size distribution. The losses can be different each second or for each vehicle or setup.

It should also be emphasized that the vehicle is not a constant source of particles or gases (inherent variability). The particulate emissions can fluctuate in terms of concentration (as in gases), but also in terms of size and chemical composition. The variability of the vehicle emissions should not be included in the uncertainty of the PN systems. Thus, it is necessary to compare the PN systems always simultaneously (measuring in parallel) in order to have relative differences, independent of the emission levels of the vehicle.

The following sections will describe how to calculate the uncertainty of the PMP system sampling from the dilution tunnel and the PEMS from the tailpipe. Even though not shown in Fig. 1, the uncertainty of PEMS can be estimated the same way for the case measuring on-board a vehicle during an on-road trip, using the appropriate input values.

### 2.2 Uncertainty of PMP system at the dilution tunnel (CVS)

The PN emissions, when measured from the full dilution tunnel (CVS) with a laboratory PMP system (PN_CVS_), are calculated as (Giechaskiel et al., 2019c, 2019f):

(1) PN_CVS_ = C_PMP_ V_CVS_ 1000 / D

(2) C_PMP_ = PCRF C_PNC_

**Fig. 2:**
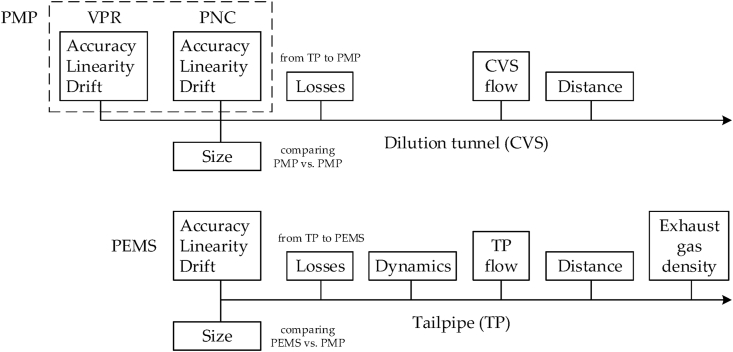
Schematic of parameters contributing to the measurement uncertainty for a PMP system at the dilution tunnel (upper part) and a PEMS at the tailpipe (lower part). CVS = constant volume sampling; PEMS = portable emissions measurement system; PMP = particle measurement programme; PNC = particle number counter; TP = tailpipe; VPR = volatile particle remover.

Where C_PMP_ is the average concentration of the PMP system (p/cm^3^), C_PNC_ is the average concentration of the PNC (p/cm^3^), V_CVS_ is the total CVS volume (L), D is the distance (km), PCRF (−) is the average particle concentration reduction factor of 30 nm, 50 nm, and 100 nm of the VPR. V_CVS_ is calculated by summing the CVS flow rate Q_CVS_ for the duration of the test. The Q_CVS_ is constant during a test or varies in a small range (<1%), and any change is taken into account the total volume V_CVS_. Fig. 2 summarizes the contributing parameters to the measurement uncertainty of a PMP system at the dilution tunnel with a causal type diagram. Fig. 2 also gives the parameters influencing the uncertainty of a PEMS at the tailpipe, which will be described in the next section.

The uncertainty of the concentration measured by the PNC, σ(C_PNC_), depends on the accuracy (k factor), σ(C_acc_), the linearity during the calibration, σ(C_lin_), and the drift that has occurred since the calibration, σ(C_drift_). All uncertainties are relative uncertainties (σ) unless otherwise specified.

(3) σ^2^(C_PNC_) = σ^2^(C_acc_) + σ^2^(C_lin_) + σ^2^(C_drift_)

This equation (error propagation) assumes independent errors. Although this might not be always true (for example, a drift might have an effect on the linearity), their dependency should be small and the covariance terms can be neglected.

The uncertainty of the PCRF of the VPR, σ(PCRF_acc_), depends on the accuracy of the reference instrument (usually a PNC_Ref_), the linearity σ(PCRF_lin_), and the drift that has occurred since the calibration σ(PCRF_drift_). The accuracy of the reference instrument (e.g. PNC_Ref_) can be estimated by Eq. (3) above without considering drift, because it is a reference instrument that is regularly controlled: σ(PCRF_acc_) = σ(PNC_Ref_). Typically, all these errors are independent to each other.

(4) σ^2^(VPR) = σ^2^(PCRF_acc_) + σ^2^(PCRF_lin_) + σ^2^(PCRF_drift_)

The measurement uncertainty of the PMP system σ(C_PMP_) can be calculated as:

(5) σ^2^(C_PMP_) = σ^2^(VPR) + σ^2^(C_PNC_) + σ^2^(size)

The first two terms of Eq. (5) give the calibration uncertainty, which is using specific particle sizes. During emissions measurements the size of particles is not known. Even though the PCRF uncertainty has been already taken into account, the mean PCRF used for the calculations is based on three sizes (30 nm, 50 nm, 100 nm) and assumes that the measured size distributions peak around 50 nm. The size uncertainty σ(size) considers the influence of the size distribution of the emitted particles on the results due to the less than unity efficiency at small sizes. As the size is unknown during the measurements and can also vary during a test, typical size ranges emitted from vehicles have to be considered. The influence of the incomplete removal of volatile particles is included in the size uncertainty, as long as there are no volatile artefacts (i.e. re-nucleation of volatile particles). Incomplete removal of the nucleation mode is considered a measurement error and is out of the scope of this paper. Such errors can influence the result by orders of magnitude. Here it is assumed that any remaining nucleation mode particles are below the lower detection size of the PN system.

The uncertainty of the PN measurement from the CVS with a PMP system, σ(PN_CVS_), can be calculated as:

(6) σ^2^(PN_CVS_) = σ^2^(C_PMP_) + σ^2^(V_CVS_) + σ^2^(D) + σ^2^(losses),

Where σ(D) is the distance uncertainty. The losses uncertainty, σ(losses), takes into account the particle losses that take place between the vehicle tailpipe and the dilution tunnel (L_TP-CVS_) and between the dilution tunnel and the instrument (L_CVS-PMP_). The main losses include agglomeration, diffusion and thermophoresis (Isella et al., 2008). The concentrations at the dilution tunnel are already low and the temperature is around 23 °C (even when the vehicle test temperature varies between −7 °C and +30 °C), thus no additional agglomeration or thermophoretic losses are expected at the tube between the dilution tunnel and the instrument (only diffusion). Thus, L_CVS-PMP_ can be considered very low (<2%) and the main contributor is L_TP-CVS_. Note that the losses inside the PN system are taken into account with the accuracy of the VPR and the PNC during calibration and with the size uncertainty. Note also that the effect of the environmental conditions σ(boundaries) is assumed to be negligible because the conditions are typically well controlled. For example, the test cell temperature varies only a few degrees during the test and the instruments are placed in temperature conditioned rooms.

### 2.3 Uncertainty of PEMS or PMP system at the tailpipe

The PN emissions when measured from the tailpipe with a PEMS (PN_TP_), are calculated as (Giechaskiel et al., 2019c, 2019f):

(7) PN_TP,i_ = 10^6^ C_PEMS,i_ Q_exh,i_ / ρ_exh,i_,

(8) PN_TP_ = Σ PN_TP,i_ / D

Where C_PEMS,i_ is the instantaneous concentration of the PEMS (p/cm^3^), Q_exh,i_ is the instantaneous exhaust flow rate (kg/s), ρ_exh_ is the density of the exhaust (kg/m^3^), and D is the distance (km). The same equations apply regardless whether the test is conducted in the laboratory or on the road.

The uncertainty of the concentration measured by the PEMS, σ(C_PEMS_), depends on the accuracy during the calibration σ(C_acc_), the linearity σ(C_lin_), and the drift that has occurred since the calibration σ(C_drift_). In addition, the size σ(size), and the boundary conditions uncertainty σ(boundaries) should be taken into account. The boundary conditions consider environmental conditions (e.g. temperature, pressure, relative humidity) that can influence the instrument. For on-road tests, vibrations are also included in this category. For laboratory testing, where the environmental conditions are constant, the boundary conditions uncertainty is negligible.

(9) σ^2^(C_PEMS_) = σ^2^(C_acc_) + σ^2^(C_lin_) + σ^2^(C_drift_) + σ^2^(size) + σ^2^(boundaries),

The first three terms of Eq. (9) determine the calibration uncertainty which is done with known particle sizes. During testing the particle size is not known. The size uncertainty σ(size) takes into account the decreasing detection efficiency at small sizes (for both condensation particle counters (CPCs) and diffusion chargers) and increasing efficiency at large sizes (for diffusion chargers). Furthermore, this uncertainty takes into account the different response functions of the instruments for different morphology or chemical composition of the particles.

Assuming that the uncertainties of the signals of PEMS, σ(C_PEMS_), and exhaust flow, σ(Q_exh_), remain the same every second (or taking the maximum uncertainty values), then the final uncertainty, σ(PN_TP_), can be estimated from the equation:

(10) σ^2^(PN_TP_) = σ^2^(C_PEMS_) + σ^2^(Q_exh_) + σ^2^(ρ_exh_) + σ^2^(D) + σ^2^(dynamics) + σ^2^(losses)

Where σ(D) is the distance uncertainty and σ(ρ_exh_) is the exhaust gas density uncertainty.

Currently all exhaust flow meters are based on the Pitot principle (Fonseca González et al., 2016), which determines volumetric flow. Corrections for the exhaust gas temperature and pressure are needed to refer to “normal” conditions (i.e. 0 °C and 101.3 kPa). The exhaust gas density is used to convert the volumetric flow to mass flow. The σ(Q_exh_) takes into account the calibration uncertainty of the flow meter, and of both pressure and temperature sensors against laboratory standards. As the calibration is done under controlled ambient conditions (around 20 °C), the possible deviations at high temperatures encountered during vehicle testing are based on theoretical equations and the experimental verification is limited. The rapid changes of temperature and any delays in the response of the temperature sensors are taken into account in the dynamics uncertainty. The density uncertainty σ(ρ_exh_) takes into account the unknown composition of the exhaust gas. Although a constant value from the tables in the regulation is used, the actual density can be different due to e.g. variations in carbon, hydrogen, oxygen content.

The losses uncertainty, σ(losses), considers agglomeration, and thermophoresis that take place at the inlet of the PEMS (L_TP-PEMS_) because these losses are not taken into account during the calibration of the PEMS at ambient temperature. As with the PMP systems, it is assumed that there is no interference from volatile particles.

The dynamics uncertainty, σ(dynamics), takes into account the time misalignment between Q_exh_ and C_PEMS_ and the different time responses of the two signals. Note that when comparing two PEMS at the tailpipe, Eq. (9) is valid only when concentrations are compared (p/cm^3^), and gives a conservative value of their expected differences. In reality the “weighing” of the concentrations with the exhaust flow rate and the influence of the dynamics should be considered to have the complete picture of expected differences between different PEMS. Thus, Eq. (10) should be used instead.

Eq. (10) assumes that the effects of the dynamics and particle losses are random and not systematic. If it is known *a priori* that a specific system has a bias due to its characteristic, then the bias could be taken into account by adding it and not considering the error propagation rule. This could be for example the case of a system with known high or low thermophoretic losses compared to other systems. However, there are many approaches in the market (e.g. direct cold or hot dilution or use of a sampling line to the instrument) to draw conclusions about bias of a specific approach.

The uncertainty of a PMP system at the tailpipe can be calculated from Eq. (10), replacing the uncertainty of PEMS Eq. (9) with the uncertainty of PMP Eq. (5). The dynamics and particle losses may be slightly different, depending on the temperatures and lengths of the sampling tubes.

An important note is that the instrument uncertainty is given by Eq. (5) (PMP) or Eq. (9) (PEMS) only when instruments are sampling from the dilution tunnel, where the sample is at ambient temperature, the flow is constant and the dynamics play minimum role. When instruments sample from the tailpipe, where the sample conditions vary, then Eq. (10) gives a better estimate of uncertainty (i.e. instrument and method uncertainty).

The previous equations assume that the PN systems are measuring in parallel (i.e. identical sample properties). For ISO/IEC 17025 a laboratory needs to assess the variability of the sample as well. In this case contributing parameters such as the cooling of the fan, the road load determination, the pre-conditioning of the vehicle, the driver, the fuel quality etc. should also be taken into account. This can be done either with repeatability or reproducibility measurements (Type A), or theoretical estimations (Type B). The sample uncertainty can be kept small by controlling all influencing parameters. Nevertheless, significant contribution is expected when the vehicles have particulate filters as the emission levels depend on their fill state (Giechaskiel et al., 2008b). Appropriate preconditioning can also minimize this effect.

### 2.4 Uncertainties input values

The values that were used for the uncertainty calculations are described below. They cover a PMP system (>23 nm or >10 nm) at the dilution tunnel or the tailpipe under laboratory conditions, and a PEMS (>23 nm or >10 nm) at the tailpipe during on-road trips. Two scenarios were examined for each case (instrument/location combination): maximum uncertainty and typical uncertainty. The maximum values were based on the maximum permissible error of the technical specifications required in the legislation or on “worst” cases based on experimental data from the literature. They include a coverage factor of k = 2 (i.e. 95% coverage), assuming that the probability distributions are normal, thus they are expanded uncertainties. The typical values were based on experimental data from commercial systems. Unless specified otherwise, the same values were applied for both 10 nm and 23 nm systems. The values are representative for PN systems measuring from approximately 6 × 10^10^ p/km or p/kWh to 6 × 10^12^ p/km or p/kWh, i.e. close to the current emission limit (6 × 10^11^ p/km or p/kWh). Lower emissions are close to the background (zero) levels of the systems, so the relative uncertainty increases. For example, with zero levels of 5000 p/cm^3^, the zero level can be up to 6 × 10^9^ p/km for light-duty vehicles and 3 × 10^10^ p/kWh for heavy-duty vehicles, depending on the engine displacement and the route. This is already a 10–50% contribution to the final result for emission levels of 6 × 10^10^ p/km or p/kWh. Higher levels than 6 × 10^12^ p/km or p/kWh will have higher uncertainty due to particle losses (agglomeration).

*PNC accuracy:* The accuracy was taken as the typical uncertainty of the reference system that is used for the calibration. For the PNC calibration, typically electrometers are used with a combined uncertainty of 2–5% (Fletcher et al., 2009; Högström et al., 2014), and even 1% expanded uncertainty had been reported (Yli-Ojanperä et al., 2012). A conservative value of 5% was assumed and 3% as typical value.

*PNC linearity:* A 10% uncertainty was assumed for PNCs based on the maximum permitted error at each calibration point (±10% allowed in the regulation), but 5% was assumed as a typical value (Giechaskiel and Stilianakis, 2009). This error will be ±5% in future due to the reduction of the permitted tolerances at the 10 nm regulation (Lahde et al., 2020). Thus, 5% uncertainty was assumed for the 10 nm systems, with 3% as typical value.

*PNC drift:* During a test, the drift should be negligible. However, the PN calibrations are conducted yearly or every 6 months. For PNCs a drift up to 20% has been reported after one year (Giechaskiel and Bergmann, 2011). Since a 6-month interval check is required (and a monthly flow check), a 10% drift was assumed. In practice this drift is much smaller because new PNC models have internal control of drift (e.g. pulse height control of the detected particles) (Scheckman et al., 2013). Typical drift values should be less than 5%. This value was assumed and includes drift due to degradation of the PNC saturators and/or clogged flow orifices.

*VPR accuracy:* For the VPR calibration (PCRF), two reference PNC_Ref_ are used upstream and downstream of the VPR simultaneously (or one PNC_Ref_ used both upstream and downstream, alternatively). The uncertainty was taken as the square root of the uncertainty of two (or one) reference PNC_Ref_. The uncertainty of the reference PNC_Ref_ was calculated assuming 5% accuracy and 3% linearity uncertainties. The linearity uncertainty of the reference PNC_Ref_ was considered in the uncertainty, because the calibration takes place at both high (upstream measurement) and low (downstream measurement) concentration levels. A low linearity uncertainty value was considered (3%), because the reference instruments are supposed to be well controlled and calibrated.

*VPR linearity:* For the VPR, there is no linearity uncertainty because each PCRF point is calibrated separately. Nevertheless, a 3% uncertainty was assumed to take into account the higher calibration uncertainty at high PCRFs (dilutions) due to the low particle concentrations during the calibration. The 3% is based on calibration certificates of reference PNC_Ref_ used for calibrations.

*VPR drift:* The PCRF of the VPR could have some drift due to orifices’ contamination, mass flow controllers’ drifts etc. (depending on the system). Based on the regulation, at the annual calibration, an instrument does not have to be re-calibrated if the calibration constants are within 10%. A 5% drift was assumed, as there are no drifts reported in the literature. This value is also in agreement with our internal data, where no drift was noticed (differences within 2% over 5 years of two units), and the variability was within the expected calibration accuracy.

*PEMS accuracy:* The PEMS efficiency is calibrated by comparing the PEMS with a reference PNC_Ref_, which typically has an accuracy around 5%. However, the technical requirements allow 15% tolerance. Thus, a 15% accuracy uncertainty was considered.

*PEMS linearity:* For PEMS a 15% uncertainty was assumed, the maximum allowed during the linearity checks.

*PEMS drift:* There is no info regarding long term drift of PEMS, so a 10% drift was assumed.

*Boundary conditions:* The effect of the boundary conditions (e.g. temperature, pressure, relative humidity, vibrations) was assumed to be 0% (i.e. there is no influence). This assumption is valid for systems measuring in the laboratory, where the conditions are controlled and stable. For on-road tests, this assumption is based on limited experimental data, showing that the instruments still respect their accuracy specifications (Valverde et al., 2020). This assumption will be discussed in the “Results and Discussion” section.

*Distance:* The laboratory uncertainty (0.4%) was based on the maximum speed error of the chassis dynamometer that is permitted in the regulation (0.08 km/h) applied to the regulated cycle WLTC (worldwide harmonized light vehicles test cycle) (23.2 km). For on-road tests, a 4% error was assumed, the maximum allowed in the regulation: difference between GPS (global positioning system) or ECU (electronic control unit) and reference distance from a map. For heavy-duty applications, instead of distance, work is used in the calculations. According to Regulation 582/2011, the load from OBD (on-board diagnostics) is accurate within 7–10%. Assuming a small error for the engine revolutions, the maximum uncertainty for the work is 10%.

*CVS flow:* For laboratory measurements, the CVS flow rate is accurate within 2%. This is confirmed weekly with a propane check. Thus, a 2% CVS flow uncertainty was applied.

*Exhaust flow:* For the tailpipe calculations or for the on-road tests, the exhaust flow should be accurate within 2–5%, but based on recent studies this uncertainty is closer to 7.5% (Giechaskiel et al., 2021). Thus, a 7.5% uncertainty was used at the calculations, but 5% for the typical case in the laboratory.

*Density:* The density of the fuel used in the laboratory is typically known because either reference fuel is used or market fuels that are chemically analyzed. Thus uncertainty 0% was used. For on-road tests the properties of the fuel can vary within the boundaries of the fuel directives and not always chemical analysis is conducted. The difference of the fuel density for various cases was <1%, and thus an uncertainty of 1% was used.

*Dynamics:* Due to the different response time of the exhaust flow meter and the PN systems, their peaks do not coincide resulting in a measurement error. There are studies that have tried to reconstruct the original signal but this has also an error and is not allowed in the regulation. Some studies estimated the uncertainty of the dynamics by misaligning the exhaust flow and the PN signal by ±1 s (Giechaskiel et al., 2019c; Hawley et al., 2004; Mohanta et al., 2014). There was no tendency of the influence and the results were typically within ±5%, reaching in some cases ±10%. These values are based on tests conducted in the laboratory with typical type approval cycles. There is lack for hybrid vehicles or on-road trips. For this reason, on road tests were re-evaluated misaligning ±1 s the PN signal compared to the exhaust flow signal. Typical RDE tests were conducted but also tests with dynamic driving exceeding the boundaries of the RDE regulation. The PEMS was based on diffusion charger principle, which has faster response than the CPC-based systems, thus the results are more sensitive to misalignment. The results for various vehicles with only internal combustion engines, or hybrids (plug in or non plug-in) are summarized in Table 1. The mean of the differences of the misaligned results were on average 1–3% lower than the aligned results, but 4–5% for the dynamic cycles. The standard deviation of the differences was 2.6% for the vehicles with only internal combustion engine (ICE), but 6.6% for the hybrids. The values were 7.5%–13% for the dynamic tests. Since the misalignment cannot be more than 0.5 s for properly calibrated systems, the previously reported values contain a factor of 2 and can be considered as expanded uncertainties. We repeated the analysis using the 10 Hz signals and the 1 s misalignment gave the same results. However, the 0.5 s results were usually lower with the 10 Hz signals. Nevertheless, for most practical applications only the 1 Hz signals are available or used, so we used these results as worst case. For the analysis a 6.5% value was used (i.e. including hybrid vehicles) and 3% as a typical value, representative for vehicles with internal combustion only. The uncertainty with dynamic driving will be discussed in the Discussion section. The values are higher than for NO_x_, which for the same tests were 2.5% (only ICE), 5.0% (ICE dynamic, hybrids), and 7.5% (hybrids dynamic). The reason is the wider range of PN measurements. No dynamics were considered for the dilution tunnel because the flow is constant during a test and an average value is used at the end of the measurement (Eq. (6) and Fig. 2).

**Table 1:** Effect of ±1 s time-misalignment on PN emissions on both urban parts and total trips. All RDE compliant tests, two different routes for each vehicle. Dynamic means the same routes but exceeding the dynamicity limits of the RDE regulation (i.e. non-compliant RDE trips).

Type	# vehicles	# tests	Emissions (min-max)	Mean	Stand. Dev.
ICE	5	10	1.8 × 10^09^–9.7 × 10^11^	−0.7%	2.6%
ICE dynamic	5	8	6.3 × 10^09^–1.9 × 10^12^	−3.6%	7.2%
Hybrid	4	13	3.8 × 10^10^–1.3 × 10^12^	−2.6%	6.6%
Hybrid dynamic	4	6	1.4 × 10^11^–2.1 × 10^12^	−5.0%	12.9%

ICE = internal combustion engine (only).

*Particle losses:* This uncertainty considers the additional particle losses that are not taken into account during the calibration of the PMP system and PEMS. These losses can take place at the sampling tube from the tailpipe to the PEMS (L_TP-PEMS_), at the tube between the vehicle and the CVS, and the CVS itself (L_TP-CVS_), or at the sampling tube from the dilution tunnel to the PMP system (L_CVS-PMP_) (see Fig. 1). The PN regulations permit dilution at the sampling point or a sampling tube with residence time up to 3 s. For PEMS connected at the tailpipe, this tube must be heated to >100 °C. The dilution can be at ambient temperature or heated. The combinations of possible losses are many and are instrument and setup specific. For this reason, a simplified approach was followed. The L_CVS-PMP_ were assumed to be only due to diffusion (2%), because the exhaust gas is already diluted and is at ambient temperature. Note that the PCRF calibration of the VPR does not include the losses in this tube, and for this reason they have to be considered separately. The L_TP-PEMS_ and L_TP-CVS_ were assumed to originate from diffusion, agglomeration and thermophoresis. The losses in the tube to the dilution tunnel or the tube to the PEMS were estimated with the same equations and assumptions, because the residence times and wall temperatures are quite similar (≤3 s and 70–100 °C). The diffusion losses in the tube from the vehicle to the dilution tunnel, or from the tailpipe to the PEMS, were assumed to be 2% each. The thermophoretic losses were estimated based on typical exhaust gas temperature profiles and assuming cooling to 100 °C (for both cases: until the entrance of the PEMS or until the mixing at the dilution tunnel) (Giechaskiel et al., 2012a). This is the worst case, because in reality the final temperatures could be higher. Furthermore, there are concepts with direct dilution at the tailpipe that minimize the thermophoretic losses. The agglomeration losses were based on various particle number concentration profiles, typical for emission levels up to 1 × 10^12^ p/km (light-duty) and 1 × 10^12^ p/kWh (heavy-duty). These losses were compared with an “ideal” system that would have the minimum losses (i.e. direct heating, dilution and counting at 350 °C, thus no diffusion or agglomeration losses, and minimum thermophoretic losses) and their differences were taken as representative values. Table 2 summarizes the results. For light-duty vehicles the losses were up to 14% mainly due to agglomeration at cold start. Thermophoresis played a small role at high speeds when the particle concentrations were also high. For heavy-duty vehicles the losses were up to 25% for CNG (compressed natural gas) fueled vehicles and 19% for diesel fueled vehicles. The agglomeration was negligible, except for a few CNG cases. The thermophoretic losses contributed up to 20% due to the “cooling” of the exhaust gas to 100 °C at the sampling tube of PEMS.

**Table 2:** Examples of estimation of particle losses for light-duty and heavy duty vehicles at the laboratory with type approval cycles or on the road with in-service conformity cycles. For max. losses a range is given because they refer to different cases/vehicles examined.

Category	T_max_ (°C)	PN_max_ (p/cm^3^)	Max. losses (%)	Ideal (%)	L_TP-PEMS_ (%)
Light-duty					
DPF (lab)	230	5 × 10^6^	0–3%	0%	3%
GDI/PFI (lab)	260	7 × 10^7^	3–14%	0%	14%
CNG (lab)	225	3 × 10^7^	4–10%	0%	10%
Heavy-Duty					
DPF (lab)	390	9 × 10^5^	16–19%	0%	19%
CNG (lab)	580	6 × 10^6^	10–24%	6%	18%
DPF (on-road)	270	8 × 10^7^	10–13%	0%	13%
CNG (on-road)	500	1 × 10^8^	24–32%	7%	25%

CNG = compressed natural gas, DPF = diesel particulate filter; GDI = gasoline direct injection; PFI = port fuel injection.

It should be emphasized that the maximum value of 25% is applicable only to CNG heavy-duty vehicles with sampling downstream of the aftertreatment devices, but close to the engine. Even in this case an ideal system would have 7% thermophoretic losses, thus the additional PEMS losses would be 18%. Thus, for heavy-duty applications a 19% value could be used, while for light-duty applications 14%. For the PMP system at the dilution tunnel, as the laboratory setups in Europe are similar, a 10% value was used (i.e. the difference of the expected losses at different laboratories). In other words, the results of different PMP systems should have smaller influence of the losses due to the similar setups. However, the differences between PEMS (or PEMS vs. PMP system) might be higher due to the different technical solutions permitted and a 14% value was used for light-duty vehicles.

Although the tailpipe measurement with PMP systems is not allowed in regulation at the moment and, thus, the technical solutions have not been defined yet, it was assumed that either direct cold dilution or hot dilution at 150 °C could be both allowed as commercial solutions. The sampling tube to the diluter would also be shorter and heated to 150 °C. Thus, the losses of the PMP systems at the tailpipe were assumed to be 12%, a value between the maximum calculated losses for PEMS (14%) and CVS (10%) solutions.

For 10 nm systems there are not many data available to do a similar assessment. The thermophoretic losses are almost size independent, and for the same concentration levels, similar agglomeration losses are expected. The diffusion losses will be slightly higher, and a constant +2% was added to the losses, representing a decrease of 15 nm on the mean size.

**Fig. 3:**
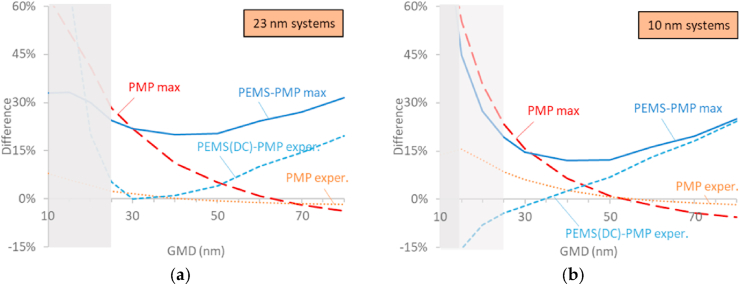
Simulated differences between PMP systems and PEMS using the maximum permissible tolerances (max) or based on experimental data (exper.): (**a**) 23 nm systems; (**b**) 10 nm systems. At the grey area the systems measure <50% of the true particle concentration. The transparent grey area is the area that 10 nm systems measure >50% of the true particle concentration, but 23 nm systems measure <50%. Details in the text and in the Appendix. DC = diffusion charger; GMD = geometric mean diameter; PEMS = portable emissions measurement system; PMP = particle measurement programme system.

*Size:* The size uncertainty includes the effect of the unknown size distribution, but also the flexibilities allowed in the regulation, because ranges of permitted efficiencies are prescribed. It is not possible to derive this uncertainty comparing with the “true” inlet concentration, because the technical requirements (e.g. PNC counting efficiency, or PEMS efficiencies) result in different “measured” concentrations. Thus, the size uncertainty of PMP systems was estimated by comparing the systems fulfilling the upper and lower limits of the technical requirements in the regulation when measuring aerosol with size distributions of different geometric mean diameters (GMDs) (Fig. 3). For PEMS, the size uncertainty was estimated by comparing them to PMP systems, which are supposed to be more accurate and are still the reference systems. Furthermore, systems with typical efficiencies, based on experimental data were compared and the results are plotted in Fig. 3. The details of the simulated system efficiencies can be found in the Appendix. The 23 nm systems measure >50% of the “true” inlet concentration when the GMDs are larger than 25 nm (Table A3), thus inlet concentrations with lower GMDs will have a big error on the absolute concentration, and in any case they are out of the scope of the regulation as the targeted cut-off size is 23 nm. The 10 nm systems measure >50% of the “true” inlet concentration at GMDs >15 nm. Thus different size ranges for the 23 nm and 10 nm systems should be considered. The size range that was considered for the 23 nm systems was 25–70 nm, based on experimental data from various studies (e.g. reviews (Giechaskiel et al., 2012b; 2019b) or recent studies (Bainschab et al., 2020)). A size range from 15 nm was examined for the 10 nm systems.

Beginning with the 23 nm PMP systems (Fig. 3a), the maximum difference decreases from 28% at 25 nm to ±5% at GMDs 50–80 nm (PMP max). The expected effect for commercial PMP systems is within ±5% (PMP exper.). The results are similar with other studies that ran similar simulations (Giechaskiel and Martini, 2014; Lahde et al., 2020). The maximum difference between PEMS (overestimating) and PMP systems (underestimating), can be 20% when the size distributions have GMDs 40–50 nm, 25% at 25 and 70 nm GMD and 30% at 80 nm (PEMS-PMP max), in agreement with other theoretical studies (Giechaskiel et al., 2019a). Note that this difference includes size uncertainty but also calibration flexibilities due to the range of permitted efficiencies. Similar differences are expected between PMP systems and PEMS having condensation particle counters (CPC) as detectors. The expected differences of PMP systems and diffusion charger based PEMS are within ±5% up to 50 nm, and increase to 20% at 80 nm GMD (PEMS(DC)-PMP exper.). Up to this size range there is no particular influence of the high efficiencies of diffusion charges at large sizes.

The results for the 10 nm systems are similar (Fig. 3b). The PMP systems differ 23% at 25 nm, but can exceed 50% at 15 nm GMD (PMP max). For market PN systems the expected differences are <15% even when small sizes are measured (PMP exper.). The maximum difference between PEMS and PMP systems are within ±25% for GMDs between 20 nm and 80 nm (PEMS-PMP max), and reaches 45% at 15 nm GMD, similar to other simulations (Giechaskiel et al., 2019e, 2020a).

Based on the previous simulations, and assuming that the majority of the GMDs is in the 25–70 nm range, a 25% size uncertainty was assumed for the differences between 23 nm PMP systems or PEMS-PMP systems. For the 10 nm systems a size range of 20–70 nm was considered. Thus, a value of 36% was assumed for the differences between 10 nm PMP systems and 27% for the 10 nm PEMS-PMP systems based on a GMD of 20 nm. Note that the 10 nm PEMS size uncertainty is not too different from the 23 nm PEMS due to tight PEMS (future) specifications at small sizes.

## 3 Results and Discussion

The uncertainty equations using the input values as discussed in the previous chapter were applied for the following cases: A 23 nm and a 10 nm PMP system at the dilution tunnel (CVS) or at the tailpipe under laboratory environmental conditions and a 23 nm and a 10 nm PEMS at the tailpipe during on-road trips. Table 3 summarizes the results. Note that as the maximum uncertainty values were used, these results can be considered as expanded uncertainty with a coverage factor of 2 (i.e. confidence level of 95%). The typical values are representative for PN systems available in the market.

**Table 3:** Maximum errors for PN systems at the dilution tunnel (CVS) or tailpipe (TP). In brackets typical uncertainties, based on experimental data of commercial systems.

Component	PMP_23 CVS	PMP_10 CVS	PMP_23 TP	PMP_10 TP	PEMS_23 TP	PEMS_10 TP
	Laboratory				Road	
Accuracy PNC	5% (3%)	5% (3%)	5% (3%)	5% (3%)	15%	15%
Accuracy VPR	8% (6%)	8% (6%)	8% (6%)	8% (6%)		
Linearity PNC	10% (5%)	5% (3%)	10% (5%)	5% (3%)	15%	15%
Linearity VPR	3%	3%	3%	3%		
Drift PNC	10% (5%)	5%	10% (5%)	5%	10%	10%
Drift VPR	5%	5%	5%	5%		
Boundaries	0%	0%	0%	0%	0%	0%
Size	25% (5%)	36% (10%)	25% (5%)	36% (10%)	25% (15%)	27% (18%)
Particle losses	10% (5%)	12% (7%)	12% (5%)	14% (7%)	14% (10%) a	16% (12%) a
Dynamics	–	–	6.5% (3%)	6.5% (3%)	6.5% (3%)	6.5% (3%)
Flow b	2%	2%	5%	5%	7.5%	7.5%
Density	–	–	0%	0%	1%	1%
Distance c	0.4%	0.4%	0.4%	0.4%	4%	4%
Calibration	18% (11%)	13% (11%)	18% (11%)	13% (11%)	23%	23%
Total	32% (14%)	40% (16%)	34% (15%)	42% (17%)	39% (31%)	41% (33%)

a Values for light-duty vehicles. For heavy-duty vehicles 4% (diesel) to 9% (CNG) more.

b CVS flow for PMP and exhaust flow for PEMS.

c Chassis dyno distance for PMP and on-road distance for PEMS. For heavy-duty applications work is needed (10% uncertainty).

The laboratory PMP systems’ uncertainty was 32% (23 nm systems) and 40% (10 nm systems). However, based on experimental data and typical setups and systems uncertainty of 14% (23 nm systems) to 16% (10 nm systems) can be expected. The expanded calibration uncertainty of the PMP systems was estimated to be 18% (VPR and PNC uncertainty, without the size uncertainty), with typical values around 11%. This value can be higher, reaching 30%, when measuring size distributions with small mean diameters. The heavy-duty regulations permit measurements from proportional partial flow dilution systems. As the concept is similar to the CVS, the uncertainty should be similar.

A 23 nm PMP system measuring at the tailpipe was estimated to have uncertainty of 34% with typical values around 15%. The additional uncertainty compared to the CVS, due to higher particle losses and the dynamics of the signals, was only 2%. Note that the uncertainties of the particle losses and dynamics were not added, but were taken into account with the error propagation rule, because they were not considered a systematic effect.

The results are quite similar with other theoretical studies. The first theoretical study for a PMP system estimated a 15% uncertainty, with a coverage factor 2 (95%) (Gilham and Quincey, 2007). At that study the size uncertainty was not taken into account. Similarly, another study calculated uncertainty of 27% (Kirchner et al., 2010), but again without taking into account the size uncertainty. Both studies are close to the 18% estimated in this study. The first experimental studies comparing many instruments in parallel with many vehicles found differences of 30% over a four orders of magnitude emission levels (with coverage factor 2 of the reported ±15% differences) probably due to calibration uncertainties in the first years of implementation of the PMP protocol (Giechaskiel et al., 2008b). Nevertheless, this value is close to the maximum calculated theoretical uncertainty of 32%. Later experimental studies with diesel vehicles found differences of <20% over many orders of magnitude (Bielaczyc et al., 2012; Giechaskiel et al., 2012b; Kim and Lee, 2011), which are in line with the 14% calculated uncertainty of typical PMP systems.

The 23 nm PEMS at the tailpipe (road) uncertainty was calculated to be around 39%, with typical values around 31%. Compared to a PMP system at the tailpipe, this is a 5% higher value, but for typical systems the difference is 16% due to the higher flexibilities allowed for PEMS (typical PEMS 31% vs. typical PMP 15%).

A check for the proper operation of a PEMS is the “validation” test, where the PEMS is compared to a laboratory PMP system measuring simultaneously the exhaust of a vehicle. Based on the analysis that was conducted before, the difference (PEMS-PMP) or ratio (PEMS/PMP) has an uncertainty that is given by the error propagation rule combining Eq. (6) and Eq. (10). The losses uncertainty should be taken only once as they refer to the difference at the two locations. Similarly, the size uncertainty (PEMS-PMP) should be considered only once as it is estimated by differences to PMP systems. The combined uncertainty is 43% (39% from PEMS and 18% from PMP system), which is close to the 50% allowed in the regulation. The combined uncertainty of typical systems is 33% (31% from PEMS and 11% from PMP system). On the other hand, the conformity factor, which takes the additional uncertainty of the PEMS compared to the PMP system could be estimated by subtracting the PEMS and PMP system uncertainties. The difference gives 34% (or 29% for typical systems). This value is close, but lower compared to the 50% in the regulation (conformity factor 1.5). Note that this 34% value is a conservative upper limit because it assumes (i) that the particle losses are higher for the PMP systems at the dilution tunnel and (ii) that the efficiency curve of the PMP systems is lower at small and large sizes (i.e. underestimating compared to PEMS). Note that subtracting the complete PEMS (39%) and PMP system (32%) uncertainties would give a 22% value, but in this case the size dependency is considered twice and is correlated, so this value is underestimating their differences.

Regarding PEMS, a theoretical study estimated 50% uncertainty (Giechaskiel et al., 2019a). Experimental studies assessing PEMS found differences of 20–30% compared to PMP systems (Khan et al., 2020; Kim et al., 2017; Kondo et al., 2019; Otsuki et al., 2019). Higher differences have been reported with diffusion chargers (Giechaskiel et al., 2018c; Khan et al., 2020), but this was due to the overestimation of charged particles (Mamakos et al., 2019). When this issue was solved the differences were within 30% (Schwelberger et al., 2019). These values are in line with the 39% calculated expanded uncertainty for 23 nm PEMS (31% for typical systems). Combined the theoretical and experimental data indicate that the current permissible tolerance of 50% should be reduced, at least to 43% (the theoretical value) and the margin to at least 0.34 from 0.50 (based on the 34% value) (Giechaskiel et al., 2021).

Inter-laboratory comparison studies can give an estimation of the uncertainty. However, they include the uncertainty of the sample preparation (i.e. vehicle etc.). The variability of the vehicle can be important, especially for DPF equipped vehicles, exceeding 30% (Giechaskiel et al., 2008a; Myung et al., 2009). A study that summarized inter-laboratory exercises for gasoline vehicles reported reproducibility values of 18–44% for PMP systems (emission levels 10^11^ to 10^12^ p/km) and 40–78% for PEMS (Giechaskiel et al., 2018a). Studies with diesel vehicles reported 50% or higher (emission levels 10^11^ p/km) (Haniu and Vallaude, 2017; Myung et al., 2009). These values are higher, but in line with the 32% and 39% estimated for the PMP and PEMS, because they include the vehicle variability.

The theoretical uncertainties of the 10 nm PEMS and PMP systems were approximately 2%–8% higher compared to the 23 nm systems, due to slightly higher particle losses for smaller particles and higher size uncertainty at lower sizes (see Fig. 3). The relatively small increase of the PEMS uncertainty (2%) was due to the stricter technical requirements for the 10 nm systems. Studies with 10 nm systems are scarce as the technical requirements were only recently finalized. The limited number of studies found differences of <30% with PMP systems (Giechaskiel et al, 2019c, 2019d, 2020a); lower than the 42% estimated in this study. The 10 nm PEMS that used were prototypes but nevertheless the differences compared to 10 nm PMP systems were well within 40% (Giechaskiel et al, 2019e, 2020a), a value that is close to the 41% calculated maximum expanded uncertainty or 44% if we consider also the 18% calibration uncertainty of the PMP system.

Surprisingly, the expanded uncertainties of 10 nm PMP systems and PEMS at the tailpipe are similar (41–42%). The reason is the high size uncertainty of the PMP system due to the possible big differences of the penetration curves at small sizes. In practice though the PMP systems have typically lower uncertainty (18% vs. 33%). Nevertheless, it is important to limit the range of allowed penetrations of sub-23 nm particles for the future 10 nm PMP systems.

The theoretical model of this study can be used to calculate the uncertainty of various cases or how the measurement uncertainty could be reduced in the future. Table 4 gives examples of PEMS uncertainties for various cases (first three columns) and theoretical improvements in order to reduce the uncertainty (last three columns). The first example is a hybrid light duty vehicle driven dynamically exceeding the dynamicity boundaries of the RDE regulation. In this case the “dynamics uncertainty” was set to 13% (see Table 1) from 6.5%; the uncertainty increased from 39% to 40%. The second example assumed a 20% effect of the boundary conditions on the PEMS (e.g. an effect of temperature or altitude on the flows). The uncertainty increased to 43%. The model was applied to heavy duty vehicles (HDV), where the particle losses can be higher (19%), as well as the work to calculate the emissions in p/kWh. The uncertainty was 41%.

**Table 4:** Maximum errors for PEMS. In bold italics the value modified from the baseline (PEMS_23 road).

Component	PEMS_23 hybrid dyn.	PEMS_23 boundaries	PMP_23 HDV	PEMS_23 calibration	PEMS_23 efficiency	PEMS_23 sampling
Linearity PEMS	15%	15%	15%	***10%***	15%	15%
Drift PEMS	15%	15%	15%	***10%***	15%	15%
Drift PEMS	10%	10%	10%	***5%***	10%	10%
Boundaries	0%	***20%***	0%	0%	0%	0%
Size	25%	25%	25%	25%	***15%***	25%
Particle losses a	14%	14%	***19%***	14%	14%	***10%***
Dynamics	***13%***	6.5%	6.5%	6.5%	6.5%	6.5%
Exhaust flow	7.5%	7.5%	***4%***	7.5%	7.5%	7.5%
Exhaust density	1%	1%	1%	1%	1%	1%
Distance^b^	4%	4%	***10%***	4%	4%	4%
Calibration	23%	23%	23%	15%	23%	23%
Total	40%	43%	41%	34%	33%	37%

a Values for light-duty vehicles, except for HDV which is based on CNG.

b On-road distance, except work for HDV.

The last three columns give examples of how to decrease the uncertainty in the future: one way is to reduce the tolerances e.g. of linearity and define often checks to ensure no drift of the systems over time. These improvements could bring down the uncertainty to 34%. Another way is to reduce the tolerances of the size dependency curves for both PMP systems and PEMS and bring them as close as possible to each other. This could bring the uncertainty to 33%. Having the same thermal pre-treatment requirements (e.g. temperatures, evaporation tube characteristics) will also help as it will result in similar system losses and particle sizes reaching the detectors of the PN systems. A more advanced option would be to force the estimation of the mean size of the size distribution and correct the concentrations (Giechaskiel et al, 2019c, 2020c). Finally, the particle losses at the tailpipe should be better controlled by defining common requirements for PEMS and PMP systems (uncertainty 37%). Applying all these improvements of the last three columns would result to a 26% PEMS uncertainty. In order to reduce further the uncertainty though would need to converge PMP systems and PEMS technical requirements with strict limits.

The previous analysis showed that the presented results can have small variations depending on the case examined. However, as the changes are not very big, even when extreme cases are considered, it can be assumed that the presented values are a good estimate of the uncertainty of the PN systems. Even though for some parameters the experimental data are limited or non-existent (e.g. drift of PEMS), the previous sensitivity analysis showed that the effect is small when the uncertainty is off by <5%. It becomes significant only when an uncertainty parameter approaches the parameter with the highest value (size dependency).

## 4 Conclusions

This study calculated the measurement uncertainty of laboratory PMP systems and on-board PEMS connected to the full dilution tunnel or the tailpipe. Systems in the current regulation are counting from 23 nm, but also future systems counting from 10 nm were simulated. This is the first complete analysis of uncertainty of PN systems.

The uncertainty took into account: (i) the technical specifications in the regulations and the permissible tolerances, (ii) the size dependency of the penetration and efficiency curves, (iii) the particle losses and transformations from the vehicle to the instruments, (iv) other parameters needed for the calculation of the emissions (flows, distance), not related to the PN systems.

The technical specifications resulted in a 18% calibration uncertainty for the 23 nm PMP systems. The size dependency of the instruments could contribute another 25% to the uncertainty. The particle losses and transformations could be as high as 19% for heavy-duty CNG engines, but 14% for light-duty vehicles. For 23 nm PMP systems at the dilution tunnel the uncertainty was estimated to be 32%, but at tailpipe 35% (due to the dynamics and higher exhaust flow uncertainty). For 23 nm PEMS measuring from the tailpipe on-road the uncertainty was 39%. The values for the 10 nm systems were estimated to be 2–8% higher. The PMP systems in the market have typically half of the previously mentioned uncertainty values (around 15–20%), but the PEMS only slightly less (around 32–34%).

A sensitivity analysis for 23 nm PEMS showed that under dynamic driving with hybrid vehicles would increase only slightly the uncertainty (1%), while the environmental conditions on PEMS could increase the uncertainty. Application of the model to heavy-duty vehicles gave an uncertainty of 41% (+2%). Finally, the model showed that improving the calibration procedures and reducing the limits could bring down the uncertainty to 26%.

The results of this study can be used as an input of the uncertainty of the measurement instruments depending on their sampling location for both regulators and researchers.

## Author contributions

Conceptualization, B.G.; formal analysis, B.G.; writing—original draft preparation, B.G.; writing—review and editing, B.G., T.L., A.M., V.V., and M.C. All authors have read and agreed to the published version of the manuscript.

## Funding

This research received no external funding.

## Disclaimer

The opinions expressed in this manuscript are those of the authors and should in no way be considered to represent an official opinion of the European Commission. Mention of trade names or commercial products does not constitute endorsement or recommendation by the European Commission or the authors.

## Declaration of competing interest

The authors declare that they have no known competing financial interests or personal relationships that could have appeared to influence the work reported in this paper.

## Figures and Tables

**Table A1:** PMP (particle measurement programme) systems simulated efficiencies and limits in the regulation for the 23 nm and 10 nm systems. H refers to low losses (high efficiencies), L to high losses (low efficiencies). CE = counting efficiency; PCRF = particle number concentration reduction factor; PNC = particle number counter; VPR = volatile particle remover.

Component	PMP_23L	PMP_23H	Limit_23	PMP_10L	PMP_10H	Limit_10
PNC CE 10 nm	0%	0%	–	50%	80%	65% ± 15%
PNC CE 15 nm	0%	0%	–	91%	98%	≥90%
PNC CE 23 nm	38%	62%	50% ± 12%	99%	100%	–
PNC CE 41 nm	90%	99%	≥90%	100%	100%	–
VPR PCRF 15/100 nm	2.00	1.14	–	2.00	1.14	≤2.00
VPR PCRF 30/100 nm	1.30	1.01	≤1.30	1.30	1.01	≤1.30
VPR PCRF 50/100 nm	1.10	1.00	≤1.20	1.10	1.00	≤1.20

**Table A2:** PEMS (portable emissions measurement systems) simulated efficiencies and limits in the regulation for the 23 nm and 10 nm systems. H refers to high efficiencies, L to low. CE = counting efficiency.

Component	PEMS_23L	PEMS_23H	Limit_23	PEMS_10L	PEMS_10H	Limit_10
CE 10 nm	–	–	–	10%	50%	10%–50%
CE 15 nm	–	–	–	34%	70%	30%–70%
CE 23 nm	20%	53%	20%–60%	–	–	–
CE 30 nm	33%	90%	30%–120%	75%	100%	75%–105%
CE 50 nm	67%	100%	60%–130%	93%	100%	85%–115%
CE 70 nm	98%	100%	70%–130%	98%	110%	85%–115%
CE 100 nm	87%	130%	70%–130%	92%	120%	80%–120%
CE 200 nm	50%	196%	50%–200%	80%	200%	80%–200%

**Fig. A1:**
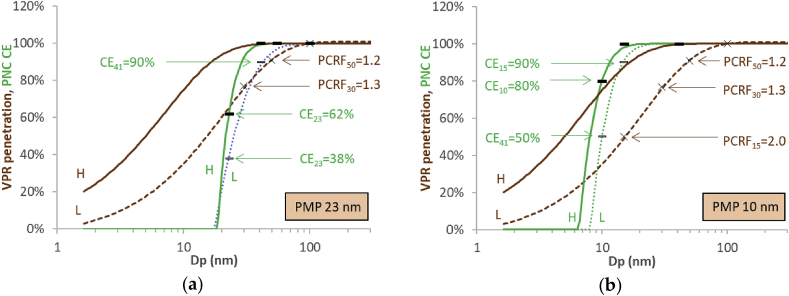
Regulation requirements and simulated VPR efficiency (penetration) normalized to 100 nm (brown) and counting efficiency (CE) of PNC corrected for the slope (i.e. normalized to the plateau region) (green). Two cases are shown: one with high efficiencies (low losses) (H) (dotted lines) and one with low efficiencies (high losses (L) (continuous lines). Symbols give the limits of the regulation: (**a**) PMP 23 nm; (**b**) PMP 10 nm. PCRF = particle concentration reduction factor; PMP = particle measurement programme; PNC = particle number counter; VPR = volatile particle remover.

**Fig. A2:**
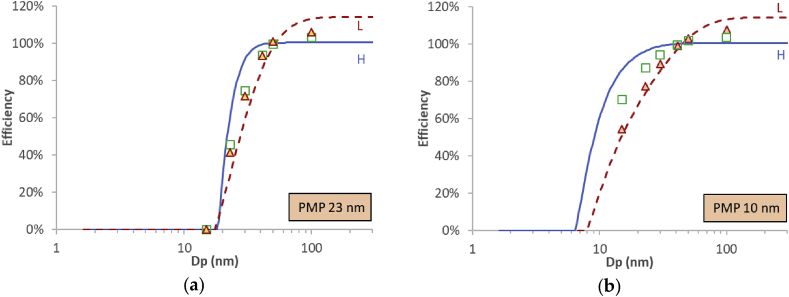
Simulated PMP efficiencies corrected with the mean PCRF and the PNC slope (i.e. normalized to the plateau region of the PNC). Two cases are shown: one with high efficiencies (low losses) (H) (blue continuous lines) and one with low efficiencies (high losses) (L) (red dotted lines) based on Figure A1. Symbols give experimental data from two instrument manufacturers: (**a**) PMP 23 nm; (**b**) PMP 10 nm. VPR = volatile particle remover; PNC = particle number counter; PMP = particle measurement programme.

**Fig. A3:**
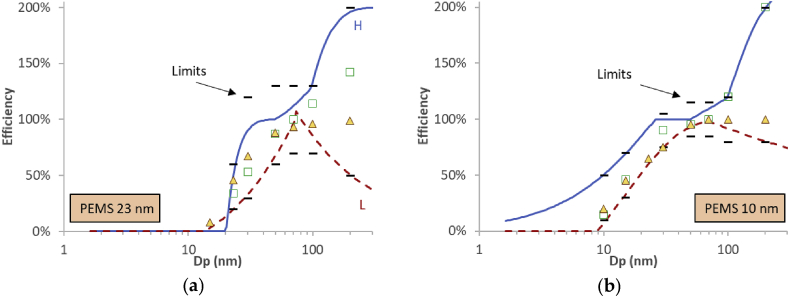
Simulated PEMS efficiencies. Two cases are shown: one with high efficiencies (H) (blue continuous lines) and one with low efficiencies (L) (red dotted lines). Symbols give experimental data from two instrument manufacturers, one based on diffusion charger (squares) and another on condensation particle counter (triangles): (**a**) PEMS 23 nm; (**b**) PEMS 10 nm. PEMS = portable emissions measurement system.

**Table A3:** Fraction of particles measured from the “true” inlet concentration by PMP (particle measurement programme) systems. H refers to low losses (high efficiencies), L to high losses (low efficiencies). T means typical. GMD = geometric mean diameter; GSD = geometric standard deviation.

GMD	GSD	PMP _23H	PMP _23L	PMP _23T1	PMP _23T2	PMP _10H	PMP _10L	PMP _10T1	PMP _10T2
10	1.7	5%	7%	7%	6%	27%	53%	40%	35%
15	1.7	16%	24%	21%	20%	49%	76%	63%	54%
20	1.7	30%	42%	38%	36%	64%	87%	76%	68%
25	1.8	45%	57%	52%	51%	74%	91%	83%	77%
30	1.8	56%	69%	63%	62%	82%	94%	89%	84%
40	1.9	73%	81%	77%	77%	91%	97%	94%	92%
50	1.9	85%	89%	86%	87%	98%	99%	98%	97%
60	2.0	91%	92%	90%	91%	101%	99%	99%	99%
70	2.0	97%	95%	94%	95%	104%	100%	100%	102%
80	2.1	99%	96%	95%	97%	106%	100%	101%	102%

**Table A4:** Fraction of particles measured from the “true” inlet concentration by PEMS (portable emissions measurement systems). H refers to high efficiencies, L to low. CPC = condensation particle counter; DC = diffusion charger; GMD = geometric mean diameter; GSD = geometric standard deviation.

GMD	GSD	PEMS _23H	PEMS _23L	PEMS _23CPC	PEMS _23DC	PEMS _10H	PEMS _10L	PEMS _10CPC	PEMS _10DC
10	1.7	6%	4%	10%	22%	54%	18%	25%	24%
15	1.7	21%	11%	24%	33%	70%	36%	44%	46%
20	1.7	39%	20%	38%	43%	81%	51%	58%	63%
25	1.8	56%	31%	49%	54%	88%	61%	67%	74%
30	1.8	69%	39%	58%	62%	94%	69%	74%	82%
40	1.9	88%	52%	70%	78%	102%	77%	83%	94%
50	1.9	102%	60%	78%	90%	109%	83%	88%	103%
60	2.0	113%	63%	83%	101%	118%	84%	91%	112%
70	2.0	123%	66%	87%	109%	124%	86%	93%	120%
80	2.1	130%	65%	89%	116%	132%	86%	94%	127%

## Appendix

The systems of the current regulation count particles from approximately 23 nm. The future regulation will require counting of particles from approximately 10 nm. This Appendix describes the estimated penetrations and counting efficiencies of the systems in order to estimate the size dependent differences between them.

### A.1 PMP Systems

A PMP system consists of a volatile particle remover (VPR) and a particle number counter (PNC) (Giechaskiel et al., 2012b).

The VPR dilutes at 150 °C and thermally treats the sample at 350 °C. The calibration requirements are that the particle concentration reduction factors (PCRFs) of 30 nm, and 50 nm have to be ≤ 1.3, and ≤1.2 times the PCRF at 100 nm. The average of 30 nm, 50 nm, and 100 nm is used as a calibration factor for the dilution and the particle losses in the VPR. The future 10 nm regulation will also have the requirement that the 15 nm PCRF has to be ≤ 2 times of the 100 nm PCRF.

The PNC is a full flow condensation particle counter (CPC) with counting efficiency of 50% (±12%) at 23 nm and ≥90% at 41 nm. The t_90_ response time is ≤ 5 s. The system is calibrated with a reference system (CPC or electrometer) at five equally spaced concentration levels (plus the zero). The difference of the PNC from the reference system has to be within ±10% at each tested point. This requirement will be reduced to ±5% in the future amendment of the 23 nm regulation, and is valid for the 10 nm instruments. The calibration factor is the slope of the linear regression of the PNC and the reference instrument. The future 10 nm regulation will require that the PNC counting efficiency will be 65% (±15%) at 10 nm and ≥90% at 15 nm (Lahde et al., 2020).

The residence time of the sample in the tube from the dilution tunnel to the PMP system has to be ≤ 3 s. The particle losses in this tube are not taken into account during calibration. Table A1 summarizes the technical calibration requirements for PMP systems.

### A.2 PEMS

The portable emissions measurement system (PEMS) has to include a heated section at a wall temperature ≥300 °C (Giechaskiel et al., 2019a). The t_10-90_ has to be ≤ 3.5 s. The residence time of the aerosol in the tube between the tailpipe and the first diluter of the PEMS has to be ≤ 3 s. The efficiency, i.e. the reading of the PEMS compared to a reference instrument, has to be within specific limits as shown in Table A2.

The PEMS (including the sampling line) has to be checked at 5 points equally distributed (plus the zero). The differences of the PEMS from the reference instrument at all points checked (except the zero point) have to be within 15% of their mean value. The linear regression results have to be: slope 1.00 ± 0.15, offset <5% of max concentration, R^2^ > 0.95.

### A.3 Simulations

Based on these technical requirements, different instruments were simulated having the minimum and maximum counting efficiencies and/or penetrations. The letter “H” was used for systems with high efficiencies (low losses) and “L” for low efficiencies (high losses). The VPR and PNC simulations are shown in Figure A1.

The efficiency of the PNC is the counting efficiency (after correcting with the slope). The efficiency (penetration) of the VPR is the inverse of the ratios of the PCRFs to the 100 nm PCRF (e.g. for 30 nm it would be 1/1.3). The used curves do not include the dependency of the material on the PNC counting efficiency (Giechaskiel and Bergmann, 2011) or the morphology on the diffusion charger efficiency (Schriefl et al., 2020). This assumption is based on the fact that practically all PNCs in the market were from a single manufacturer. It is also assumed that the calibration is done with thermally stable particles. If the particles are not stable, changes in the VPR will result in different PCRFs (Sakurai et al., 2011).

Figure A2 shows the experimental and simulated efficiencies of PMP systems. The efficiency of the PMP systems at each size was calculated as the product of the efficiency (penetration) of the VPR and the PNC. The efficiency (penetration) of the PMP system was also multiplied with the average PCRF of 30 nm, 50 nm, and 100 nm as required in the regulation. A PMP system, with low penetration (high losses), due to the average PCRF that is used, will overestimate the concentration of large particles (for this reason the efficiency is higher than 100%). Experimental data were taken from calibration certificates from the two largest instrument manufacturers in Europe (Giechaskiel et al, 2019e, 2020a; Lahde et al., 2020; Otsuki et al., 2014; Schwanzer et al., 2019; Yamada et al., 2015).

The simulated efficiencies show that the PNC efficiencies define the PMP efficiency for the currently 23 nm regulated systems. The efficiencies of the future 10 nm PM systems are determined by both the VPR and the PNC, but the VPR plays the most important role.

Figure A3 shows the experimental and simulated efficiencies for PEMS. It should be noted that the simulated PEMS efficiencies do not follow the higher or lower limits in the regulation, because the linearity has to give a slope of 1.00 ± 0.15. Thus, there must be a size that there is perfect agreement with the reference instrument (efficiency 100%) at a size >45 nm. Experimental data from (Giechaskiel et al, 2019e, 2020a; Otsuki et al., 2017).

Table A3 and A4 summarize the percentages that each system measures compared to their inlet concentration. The values are close to previous similar studies (Bainschab et al., 2020; Giechaskiel and Martini, 2014; Lahde et al., 2020).
